# Automatic Diagnosis of Schizophrenia in EEG Signals Using CNN-LSTM Models

**DOI:** 10.3389/fninf.2021.777977

**Published:** 2021-11-25

**Authors:** Afshin Shoeibi, Delaram Sadeghi, Parisa Moridian, Navid Ghassemi, Jónathan Heras, Roohallah Alizadehsani, Ali Khadem, Yinan Kong, Saeid Nahavandi, Yu-Dong Zhang, Juan Manuel Gorriz

**Affiliations:** ^1^Faculty of Electrical Engineering, K. N. Toosi University of Technology, Tehran, Iran; ^2^Department of Medical Engineering, Islamic Azad University of Mashhad, Mashhad, Iran; ^3^Faculty of Engineering, Islamic Azad University of Science and Research, Tehran, Iran; ^4^Department of Mathematics and Computer Science, University of La Rioja, Logroño, Spain; ^5^Institute for Intelligent Systems Research and Innovation (IISRI), Deakin University, Geelong, VIC, Australia; ^6^School of Engineering, Macquarie University, Sydney, NSW, Australia; ^7^Department of Informatics, University of Leicester, Leicester, United Kingdom; ^8^Department of Signal Theory, Telematics and Communications, ETS of Computer and Telecommunications Engineering, University of Granada, Granada, Spain

**Keywords:** schizophrenia, neuroimaging, EEG signals, diagnosis, deep learning

## Abstract

Schizophrenia (SZ) is a mental disorder whereby due to the secretion of specific chemicals in the brain, the function of some brain regions is out of balance, leading to the lack of coordination between thoughts, actions, and emotions. This study provides various intelligent deep learning (DL)-based methods for automated SZ diagnosis *via* electroencephalography (EEG) signals. The obtained results are compared with those of conventional intelligent methods. To implement the proposed methods, the dataset of the Institute of Psychiatry and Neurology in Warsaw, Poland, has been used. First, EEG signals were divided into 25 s time frames and then were normalized by *z*-score or norm L2. In the classification step, two different approaches were considered for SZ diagnosis *via* EEG signals. In this step, the classification of EEG signals was first carried out by conventional machine learning methods, e.g., support vector machine, *k*-nearest neighbors, decision tree, naïve Bayes, random forest, extremely randomized trees, and bagging. Various proposed DL models, namely, long short-term memories (LSTMs), one-dimensional convolutional networks (1D-CNNs), and 1D-CNN-LSTMs, were used in the following. In this step, the DL models were implemented and compared with different activation functions. Among the proposed DL models, the CNN-LSTM architecture has had the best performance. In this architecture, the ReLU activation function with the *z*-score and L2-combined normalization was used. The proposed CNN-LSTM model has achieved an accuracy percentage of 99.25%, better than the results of most former studies in this field. It is worth mentioning that to perform all simulations, the *k*-fold cross-validation method with *k* = 5 has been used.

## Introduction

Schizophrenia (SZ) is one of the most important mental disorders, leading to disruption in brain growth (Lewis and Levitt, 2002; Schmitt et al., 2011). This disorder seriously damages thoughts, expression of emotions, and also individuals' perception of reality (Elvevag and Goldberg, 2000). The reason for SZ is not fully understood, though most research has demonstrated that the structural and functional abnormalities of the brain play a role in its creation (Qureshi et al., 2019). According to the World Health Organization reports, nearly 21 million individuals suffer from such a brain disorder worldwide. The average age starting to get affected by this disorder is in youth age; in men 18 years old, and women 25 years old, and it is more prevalent among males (Sadeghi et al., 2021).

Numerous methods have been provided for automated SZ diagnosis; among these techniques, neuroimaging-based methods have a special potential for specialist physicians (Li et al., 2021; Yan et al., 2021). Generally, neuroimaging methods include various structural or functional modalities (Steardo et al., 2020; Hu et al., 2021). Structural MRI and diffusion tensor imaging-MRI are among the most important modalities of structural neuroimaging, providing important information regarding brain structure to specialist physicians (Sui et al., 2013; Lee et al., 2018; Oh et al., 2020). Contrarily, electroencephalography (EEG) (Boutros et al., 2008), magnetoencephalography (Fernández et al., 2011), functional MRI (Sartipi et al., 2020), and functional near-infrared spectroscopy (Chen et al., 2020) are the most important functional modalities of the brain. These modalities provide vital information on brain function to specialist physicians.

EEG is one of the most practical and inexpensive functional neuroimaging modalities, specifically capturing the interests of specialist physicians. In this modality, the electrical activities of the brain are recorded from the head surface with a high temporal resolution and an appropriate spatial resolution, which is influential in SZ diagnosis (Murashko and Shmukler, 2019). In addition to the mentioned merits, EEG signals regularly have various channels recorded in the long term (Murashko and Shmukler, 2019). In some cases, these reasons make specialist physicians face serious challenges in SZ diagnosis *via* EEG signals.

In recent years, various investigations have provided automated SZ diagnosis *via* EEG signals using artificial intelligence (AI) methods (Prasad et al., 2013; Shim et al., 2016; Chu et al., 2017; Alimardani et al., 2018; Devia et al., 2019; Jahmunah et al., 2019; Li et al., 2019; Naira and Alamo, 2019; Oh et al., 2019; Phang et al., 2019a,b; Aristizabal et al., 2020; Luo et al., 2020; Prabhakar et al., 2020; Shalbaf et al., 2020; Siuly et al., 2020; Sharma et al., 2021; Singh et al., 2021; Sun et al., 2021). The AI investigations in this field include conventional machine learning (ML) and deep learning (DL) methods (Khodatars et al., 2020; Shoeibi et al., 2020, 2021,a,b). The AI-based SZ diagnosis algorithm includes preprocessing sections, features extraction and selection, and in the end, classification. Feature extraction is the most important part of SZ diagnosis *via* EEG signals. In conventional ML, the extracted features from EEG signals are mainly categorized into four groups: time (Diykh et al., 2016), frequency (Faust et al., 2010), time-frequency (Madhavan et al., 2019), and non-linear (Gajic et al., 2015; Shoeibi et al., 2021a) fields. Siuly et al. (2020) used empirical mode decomposition (EMD) in preprocessing step. In the following, various statistical features were extracted from EMD subbands, and the ensemble bagged tree method was used for classification. In another study, Jahmunah et al. (2019) used non-linear features and support vector machine (SVM) with radial basis function kernel in the feature extraction and classification steps, respectively. Devia et al. (2019) have provided an event-related field features-based SZ diagnosis method *via* EEG signals. Extremely randomized trees (ERT) features were extracted from EEG signals in this effort, and then linear discriminant analysis was used in the classification step. In Prabhakar et al. (2020), statistical features of steady-state visual evoked potentials were extracted, and in the end, classification has been executed by the *k*-nearest neighbors (KNN) method. Li et al. (2019) used solitary pulmonary nodule features and SVM classification for SZ diagnosis *via* EEG signals. In another study, Shim et al. provided a new method of SZ diagnosis *via* EEG signals (Shim et al., 2016). This investigation used sensor-level and source-level features in the feature extraction step and then employed the Fisher's score for feature selection. Ultimately, the SVM method was used in the classification step, and they achieved promising results.

In conventional ML, selecting proper feature extraction algorithms for SZ diagnosis is a relatively demanding task, requiring a great deal of knowledge in signal processing and the AI field. To overcome this problem, DL-based methods have been provided in recent years for SZ diagnosis *via* EEG signals, where feature extraction operations are carried out without surveillance by deep layers (Shoeibi et al., 2021a). Shalbaf et al. (2020) define a transfer learning model for SZ diagnosis *via* EEG signals. In this study, the ResNet-18 model has been used for feature extraction from EEG signals. Besides, SVM has been used in the classification step. Some researchers have studied other convolutional network (CNN) models utilization in SZ diagnosis *via* EEG signals. CNN models have been used in Naira and Alamo (2019) and Oh et al. (2019) for SZ diagnosis, resulting in satisfactory achievements. CNN-recurrent neural network (RNN) models are an important group of DL networks and are significantly popular for their capability of various brain diseases diagnoses *via* EEG signals. In Aristizabal et al. (2020), Sharma et al. (2021), Singh et al. (2021), Sun et al. (2021), CNN-long short-term memory (LSTM) models have been used for SZ diagnosis, and the researchers have been able to achieve promising results.

**Figure 1:**
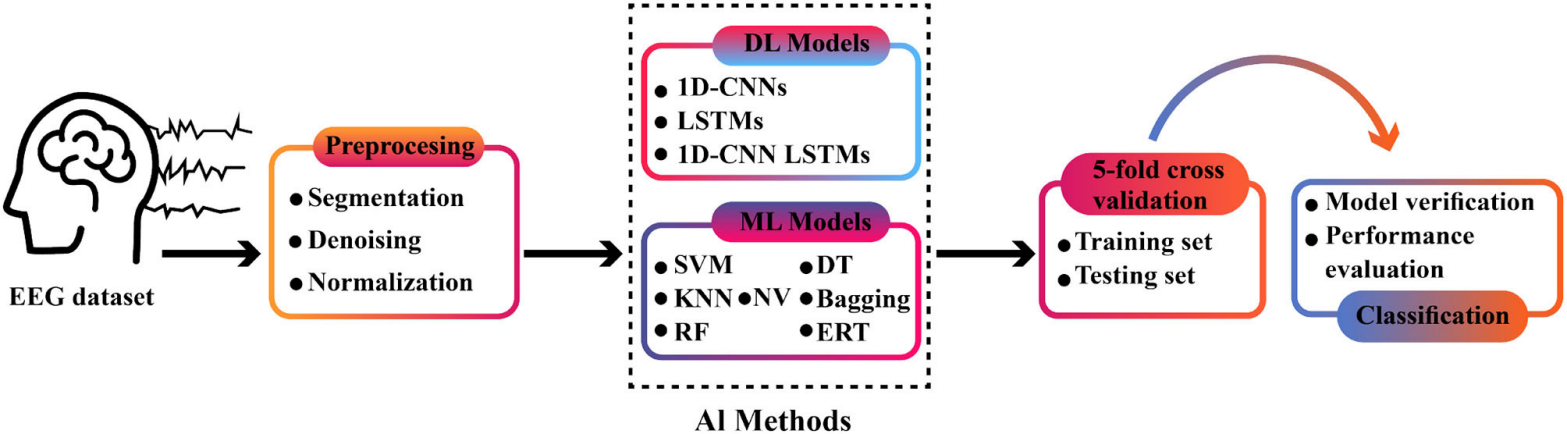
The block diagram of proposed methods.

In this paper, SZ diagnosis *via* EEG signals will be investigated by using various proposed DL and conventional ML-based methods. A summary of proposed methods is depicted in Figure 1.

In this study, the dataset of the Institute of Psychiatry and Neurology in Warsaw, Poland, is used (Olejarczyk and Jernajczyk, 2017). In the preprocessing step, the *z*-score and L2 normalization techniques will be applied to EEG signals. Next, to classify EEG signals, various conventional ML methods and DL-based proposed models will be used. The conventional ML methods employed, include various classification, SVM (Cortes and Vapnik, 1995), KNN (Cover and Hart, 1967), decision tree (DT) (Rokach and Maimon, 2007), naïve Bayes (Zhang, 2004), random forest (RF) (Breiman, 2001), ERT (Geurts et al., 2006), and bagging (Friedman, 2001) methods. Besides, the proposed DL networks include various one-dimensional (1D)-CNN, LSTM, and ID-CNN-LSTM models for executing the steps from feature extraction to classification. Generally, nine LSTM-, 1D-CNN-, and ID-CNN-LSTM-based DL methods will be investigated in this step.

In section Materials and Methods, we described our method in detail. In addition, we outline several baseline methods for comparison purposes in the same section. The statistical metrics to analyze and validate the proposed model are described in section Experiment Results. Experiment results are provided in section Limitation of Study, and some limitations of the proposed method are provided in section Conclusion, Discussion, and Future Works. Finally, a discussion, the conclusion, and future works are represented.

## Materials and Methods

This section will discuss the proposed methods for SZ diagnosis *via* EEG signals and various conventional ML and DL models. First, the proposed dataset will be examined. Then, the preprocessing method of EEG signals will be explained. In the end, conventional ML and DL models will be introduced for SZ diagnosis *via* EEG signals.

### Dataset

**Figure 2:**
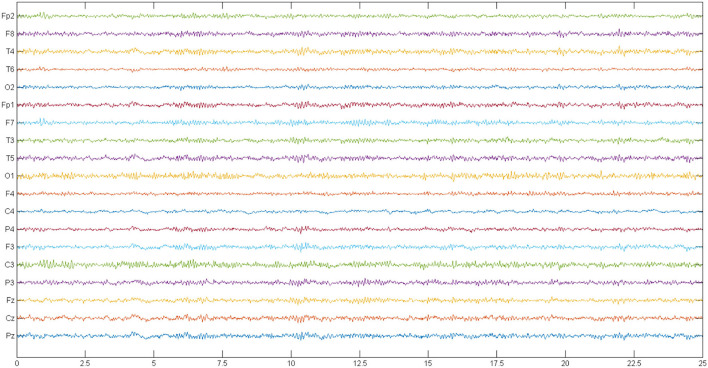
A sample frame of the EEG signals of a person with SZ. EEG, electroencephalograph; SZ, schizophrenia.

**Figure 3:**
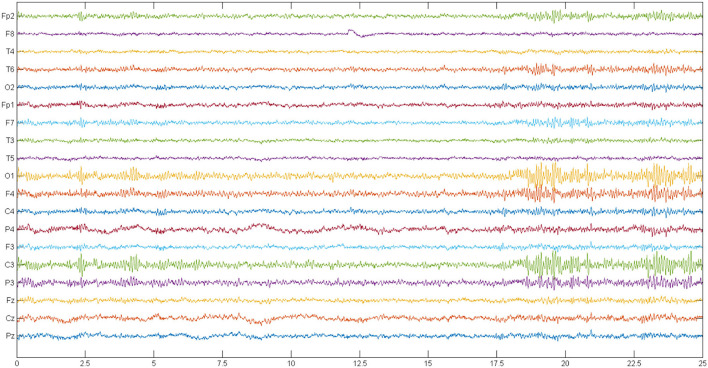
A sample frame of the EEG signals of a normal person. EEG, electroencephalograph.

This dataset includes recorded EEG signals from 14 females and males with ages between 27.9 and 28.3 years. Besides, 14 normal individuals matched with the patients in terms of age and gender were employed in this institution, and the data recording was carried out (Olejarczyk and Jernajczyk, 2017). A signal recording was performed with the eyes closed in 15 min for each case. Recording EEG signals was performed by using standard 10–20 with a sampling frequency of 250 Hz (Olejarczyk and Jernajczyk, 2017). In this study, the used electrodes include Fp1, Fp2, F7, F3, Fz, F4, F8, T3, C3, Cz, C4, T4, T5, P3, Pz, P4, T6, O1, and O2. An example of EEG signals of SZ and normal cases is depicted in Figures 2, 3.

### Preprocessing

To preprocess the EEG signals of the mentioned dataset, several steps are used. First, each 19 recorded EEG signal has been divided into overlap-free 25 s frames, each of which includes 6,250 temporal samples. Accordingly, each frame of EEG signals has 6,250 × 19 dimensions. In the following, each EEG frame has been normalized by *z*-score and L2 methods. The normalization of EEG signals helps the accuracy and performance enhancement in conventional ML and DL models.

### Conventional Machine Learning Methods

The proposed conventional ML methods are introduced in this section as a baseline for comparison purposes. The proposed algorithms include SVM (Cortes and Vapnik, 1995), KNN (Cover and Hart, 1967), DT (Rokach and Maimon, 2007), naïve Bayes (Zhang, 2004), RF (Breiman, 2001), ERT (Geurts et al., 2006), and bagging (Friedman, 2001). Each of these methods will be briefly introduced in the following.

### Support Vector Machine

Support vector machine (SVM) (Cortes and Vapnik, 1995) is an algorithm that constructs a hyperplane or set of hyperplanes in a high- or infinite-dimensional space, which can be used for classification, regression, or other tasks. Intuitively, a good separation is achieved by the hyperplane that has the largest distance to the nearest training data points of any class (so-called functional margin), since in general the larger the margin the lower the generalization error of the classifier.

#### k-Nearest Neighbors

*k*-nearest neighbor (KNN) (Cover and Hart, 1967) is a classification algorithm where some fixed and small number (*k*) of nearest neighbors (based on a notion of distance) from the training set are located and used together to determine the class of the test instance through a simple majority voting; that is, the class of the test instance is assigned the data class which has the most representatives within the KNN of that point.

#### Decision Tress

Decision trees (DTs) (Rokach and Maimon, 2007) is an algorithm that creates a model that predicts the class of an instance by learning simple decision rules inferred from the data features. The representation of a DT model is a binary tree wherein each node represents a single input variable (*X*) and a split point on that variable, assuming the variable is numeric. The leaf nodes (also called terminal nodes) of the tree contain an output variable (*y*) which is used to make a prediction.

#### Naïve Bayes

Naive Bayes (Zhang, 2004) is a supervised learning algorithm based on applying Bayes' theorem with the “naive” assumption of conditional independence between every pair of features given the value of the class variable. This means that we calculate P(data|class) for each input variable separately and multiple the results together, for example: P(class | X1, X2, …, Xn) = P(X1|class) × P(X2|class) × … × P(Xn|class) × P(class) / P(data); where P(A | B) represents the probability of A given B.

#### Random Forest

Random forest (RF) (Breiman, 2001) is an extension of the bagging algorithm where several DT classifiers are fit on various subsamples of the dataset and uses averaging to improve the predictive accuracy and control over-fitting. Unlike bagging, RF also involves selecting a subset of input features (columns or variables) at each split point in the construction of trees. By reducing the features to a random subset that may be considered at each split point, it forces each DT in the ensemble to be more different.

#### Extremely Randomized Trees

Extremely randomized trees (ERT) (Geurts et al., 2006), like RF, is an ensemble of several DT models. However, the ERT algorithm fits each DT on the whole training dataset instead of using a bootstrap sample. Like the RF algorithm, the ERT algorithm will randomly sample the features at each split point of a DT; but instead of using a greedy algorithm to select an optimal split point, the ERT selects a split point at random.

#### Bagging

Bagging (Friedman, 2001) is an ensemble classifier that fits base classifiers on random subsets of the original dataset and then aggregates their individual predictions (either by voting or by averaging) to form a final prediction. To be more concrete, in bagging, several classifiers are created where each classifier is created from a different bootstrap sample of the training dataset. A bootstrap sample is a sample of the training dataset where a sample may appear more than once in the sample, referred to as sampling with replacement.

### Deep Learning Models

This section provides various types of 1D-CNN, LSTM, and 1D-CNN-LSTM models for SZ diagnosis *via* EEG signals. Various types of the suggested 1D-CNN, LSTM, and 1D-CNN-LSTM models will be examined in the following.

#### ID-CNN Models

The higher performance of CNN models in machine vision has led them to be used in time series processing, such as medical signals, leading to successful results (Chen et al., 2019; Mahmud et al., 2021). The CNN models have important convolutional, pooling, and fully connected (FC) layers (Niepert et al., 2016; Zhang et al., 2019). In 1D-CNN models, signal time can be considered a spatial dimension, e.g., height or width of a 2D image (Goodfellow et al., 2016). 1D-CNN models are considered the important rivals of RNN architectures in time series processing. Compared to RNN models, 1D-CNN architectures have lower computational costs (Goodfellow et al., 2016). In this section, the three proposed 1D-CNN-based models are provided for SZ diagnosis *via* EEG signals.

**(A) The first version of 1D-CNN model**

The details of the first proposed 1D-CNN model are provided in Table 1. Concerning Table 1, this model includes nine different layers. The convolutional layers have 64 filters with 3 ×3 dimensions. In addition, various activation functions, e.g., ReLU, Leaky ReLU, and seLU, have been used in convolutional layers, and the related results will be compared in the Experiment Results section. Besides, a max-pooling layer has been used for decreasing dimensions, dropout layers with different rates for the prevention of overfitting, flatten layer for converting a matrix to vector, and in the end, dense layers for classification. The activation function of the final dense layer is of sigmoid type, used for binary classification.

**Table 1:** The details of the first proposed 1D-CNN model.

**Layers**	**Details Layers**	**Filters**	**Kernel size**	**Stride**	**Activation**
1	Input Data	–	–	–	–
2	Conv1D	64	3	1	ReLU
3	Conv1D	64	3	1	ReLU
4	Dropout	–	–	Rate = 0.5	–
5	Max Pooling	–	2	1	–
6	Flatten	–	–	–	–
7	Dense	100	–	–	–
8	Dropout	–	–	Rate = 0.25	–
9	Dense	1	–	–	Sigmoid

*1D-CNN, one-dimensional convolutional network*.

**(B) The second version of 1D-CNN model**

The architecture of the second proposed 1D-CNN model has three convolutional layers, and their filters' number, kernel size, and activation function have been indicated in Table 2. In this model, a convolutional layer with a kernel size of 2 has been used. Moreover, this model has four dropout layers with different rates, one flatten layer and two dense layers. The activation function of the first dense layer is of ReLU type, and the activation function of the final dense layer is for sigmoid classification.

**Table 2:** The details of the second proposed 1D-CNN model.

**Layers**	**Details Layers**	**Filters**	**Kernel Size**	**Stride**	**Activation**
1	Input Data	–	–	–	–
2	Conv1D	64	3	1	ReLU
3	Dropout	–	–	Rate = 0.5	–
4	Conv1D	64	3	1	ReLU
5	Dropout	–	–	Rate = 0.5	–
6	Conv1D	64	3	1	ReLU
7	Dropout	–	–	Rate = 0.5	–
8	Max Pooling	–	2	1	–
9	Flatten	–	–	–	–
10	Dense	100	–	–	ReLU
11	Dropout	–	–	Rate = 0.25	–
12	Dense	1	–	–	Sigmoid

*1D-CNN, one-dimensional convolutional network*.

**(C) The third version of 1D-CNN model**

According to Table 3, the third proposed 1D-CNN model consists of two convolutional layers with a similar number of filters, kernel size, and activation functions to the previous networks. This model has a max pooling layer with a kernel size of 2. In addition, it takes advantage of dropout with different rates. Similar to previous models, a flatten layer is also used in this model. This model consists of two dense layers, in which the activation functions of the first and second layers are of ReLU and sigmoid type, respectively.

**Table 3:** The details of the third proposed 1D-CNN model.

**Layers**	**Details Layers**	**Filters**	**Kernel Size**	**Stride**	**Activation**
1	Input Data	–	–	–	–
2	Conv1D	64	3	1	ReLU
3	Conv1D	64	3	1	ReLU
4	Dropout	–	–	Rate = 0.5	–
5	Max Pooling	–	2	1	–
6	Flatten	–	–	–	–
7	Dense	100	–	–	ReLU
8	Dropout	–	–	Rate = 0.25	–
9	Dense	50	–	–	ReLU
10	Dropout	–	–	Rate = 0.25	–
11	Dense	1	–	–	Sigmoid

*1D-CNN, one-dimensional convolutional network*.

#### LSTM Models

Recurrent neural networks (RNNs) are a group of DL models employed in speech recognition (Ogunfunmi et al., 2019), natural language processing (Deng and Liu, 2018), and biomedical signal processing (Vicnesh et al., 2020; Baygin et al., 2021). CNN models are of Feed-Forward types. However, the RNNs have a FeedBack layer, in which the network output returns to the network along with the next input. Because of having internal memory, RNNs memorize their previous input and use it to process a sequence of inputs. Simple RNN, LSTM, and gated recurrent unit networks are three important groups of RNNs (Goodfellow et al., 2016). In this section, various LSTM models of SZ diagnosis *via* EEG signals will be proposed.

**(A) The first version of LSTM model**

In Table 4, the details of the first proposed LSTM model consisting of six layers are presented. In this model, an LSTM layer with a kernel size of 100 is employed. Another section of the proposed LSTM architecture consists of two different layers of dropout and rate and two dense layers. In the first and second dense layers, the ReLU and sigmoid activation functions are used.

**Table 4:** The details of the first proposed LSTM model.

**Layers**	**Details Layers**	**Filters**	**Kernel Size**	**Stride**	**Activation**
1	Input Data	–	–	–	–
2	LSTM	1	100	–	–
3	Dropout	–	–	Rate = 0.5	–
4	Dense	100	–	–	ReLU
5	Dropout	–	–	Rate = 0.25	–
6	Dense	1	–	–	Sigmoid

*LSTM, long short-term memory*.

**(B) The second version of LSTM model**

In Table 5, the details of the second proposed LSTM model consisting of seven layers are presented. In this architecture, an LSTM layer with a kernel size of 50 is added to the previous model. The reason behind this is to examine the effect of adding LSTM layers on SZ diagnosis accuracy *via* EEG signals.

**Table 5:** The details of the second proposed LSTM model.

**Layers**	**Details Layers**	**Filters**	**Kernel Size**	**Stride**	**Activation**
1	Input Data	–	–	–	–
2	LSTM	1	100	–	–
3	LSTM	1	50	–	–
4	Dropout	–	–	Rate = 0.5	–
5	Dense	100	–	–	ReLU
6	Dropout	–	–	Rate = 0.25	–
7	Dense	1	–	–	Sigmoid

*LSTM, long short-term memory*.

#### CNN-LSTM Models

In CNN-RNN models, the convolutional layers are used in the first layers of the model to extract the features and find the local patterns (Goodfellow et al., 2016). Then, their outputs are applied to RNN layers. Experimentally, the convolutional layers extract the local and spatial patterns of EEG signals better compared to RNNs. Besides, adding convolutional layers to RNN allows a more accurate examination of data. In this section, various CNN-LSTM models for SZ diagnosis will be proposed.

**(A) The first version of CNN-LSTM model**

The first proposed CNN-LSTM model consists of 11 max, dropout, CNN, LSTM, flatten, pooling, and dense layers. The details of the proposed model are presented in Table 6. This architecture includes two convolutional layers; three dropout layers with different rates, one Max-Pooling layer, and one flatten layer, one LSTM layer, and finally, two dense layers with ReLU and sigmoid activation functions.

**Table 6:** The details of the first proposed CNN-LSTM model.

**Layers**	**Details Layers**	**Filters**	**Kernel Size**	**Stride**	**Activation**
1	Input Data	–	–	–	–
2	Conv1D	64	3	1	ReLU
3	Conv1D	64	3	1	ReLU
4	Dropout	–	–	Rate = 0.5	–
5	Max Pooling	–	2	1	–
6	Flatten	–	–	–	–
7	LSTM	1	100	–	–
8	Dropout	–	–	Rate = 0.5	–
9	Dense	100	–	–	ReLU
10	Dropout	–	–	Rate = 0.25	–
11	Dense	1	–	–	Sigmoid

*CNN, convolutional network; LSTM, long short-term memory*.

**(B) The second version of CNN-LSTM model**

**Figure 4:**
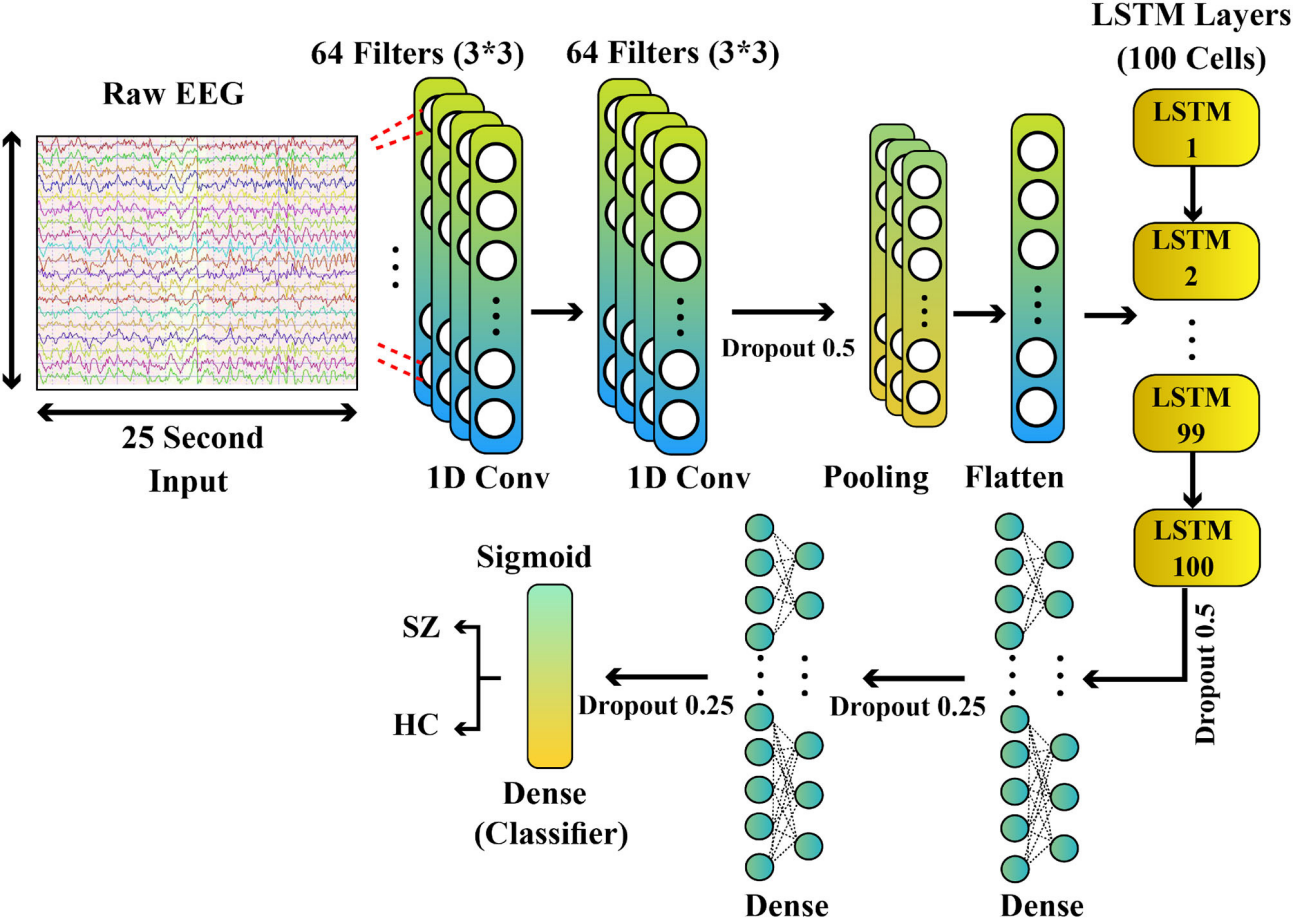
The second version of the proposed CNN-LSTM model for diagnosis of SZ. CNN, convolutional network; LSTM, long short-term memory; SZ, schizophrenia.

In this section, the second proposed CNN-LSTM model will be introduced. This network includes 13 layers, and similar to the previous model, it consists of CNN and LSTM layers whose details are demonstrated in Table 7 and Figure 4. As can be seen in Table 7 and Figure 4, the first 10 layers of this proposed model are identical to those of the previous CNN-LSTM model. The dense layer with 50 neurons and the ReLU activation function is used in the 11th layer of this architecture. The 12th layer comprises a dropout with a rate = 0.25. Ultimately, in the 13th layer, the dense layer with a sigmoid activation function for classification is employed.

**Table 7:** The details of the second proposed CNN-LSTM model.

**Layers**	**Details Layers**	**Filters**	**Kernel Size**	**Stride**	**Activation**
1	Input Data	–	–	–	–
2	Conv1D	64	3	1	ReLU
3	Conv1D	64	3	1	ReLU
4	Dropout	–	–	Rate = 0.5	–
5	Max Pooling	–	2	1	–
6	Flatten	–	–	–	–
7	LSTM	1	100	–	–
8	Dropout	–	–	Rate = 0.5	–
9	Dense	100	–	–	ReLU
10	Dropout	–	–	Rate = 0.25	–
11	Dense	50	–	–	ReLU
12	Dropout	–	–	Rate = 0.25	–
13	Dense	1	–	–	Sigmoid

*CNN, convolutional network; LSTM, long short-term memory*.

## Experiment Results

The results of the proposed methods are presented in this section. First, the simulation results obtained from conventional ML techniques for SZ diagnosis *via* EEG signals are presented and discussed. The original dataset was flattened to have only a vector per sample, and then we used the flattened dataset to train several classification algorithms using the scikit-learn library (Pedregosa et al., 2011). Namely, we studied the performance of KNNs, DTs, SVMs, and naive Bayes; and three ensemble algorithms (bagging, extremely randomized trees, and RF). The algorithms were trained using the by-default hyperparameters provided by the implementation of the scikit-learn library. Moreover, we studied the impact of z-score normalization (Cheadle et al., 2003) on the performance of the models. All the experiments were conducted in an Intel (R) Core (TM) i7-4810MQ CPU at 2.80 GHz. In Table 8, the results obtained from conventional classification algorithms for raw input EEG signals or normalized by *z*-score normalization are indicated.

**Table 8:** Performance criteria of conventional ML classifiers.

**Methods**	**Raw EEG**				**z-Score Normalized EEG**			
	**Acc**	**Prec**	**Rec**	**AUC**	**Acc**	**Prec**	**Rec**	**AUC**
KNN	57.03 ± 2.21	52.12 ± 2.66	99.80 ± 0.38	59.58 ± 0.56	55.10 ± 1.77	49.32 ± 1.42	99.80 ± 0.39	60.13 ± 1.28
DT	64.19 ± 3.08	62.49 ± 5.15	59.52 ± 5.40	63.94 ± 3.12	64.71 ± 4.12	59.28 ± 5.00	61.16 ± 5.14	64.31 ± 4.21
SVM	54.14 ± 3.97	20.77 ± 25.50	32.57 ± 39.96	54.10 ± 5.16	62.09 ± 2.75	54.72 ± 2.92	77.81 ± 2.01	63.89 ± 2.42
Bayes	62.62 ± 2.52	56.08 ± 2.76	93.21 ± 4.60	64.35 ± 2.30	59.12 ± 3.26	51.78 ± 2.38	94.81 ± 2.61	63.15 ± 2.97
Bagging	77.37 ± 3.23	81.80 ± 2.56	66.93 ± 6.13	76.91 ± 2.96	**81.22** **±** **1.74**	82.90 ± 3.76	72.02 ± 1.95	80.21 ± 1.65
RF	75.19 ± 2.19	83.60 ± 4.22	59.00 ± 3.62	74.20 ± 1.43	78.77 ± 1.55	81.23 ± 2.31	66.80 ± 2.94	77.44 ± 1.74
ERT	76.24 ± 1.84	80.64 ± 3.37	64.96 ± 2.10	75.57 ± 1.52	76.94 ± 1.81	76.29 ± 2.27	68.35 ± 3.90	75.96 ± 2.05

*AUC, area under the curve; DT, decision tree; EEG, electroencephalograph; KNN, k-nearest neighbor; ML, machine learning; RF, random forest; SVM, support vector machine. The bold values provide the highest accuracy the method compared other methods*.

**Figure 5:**
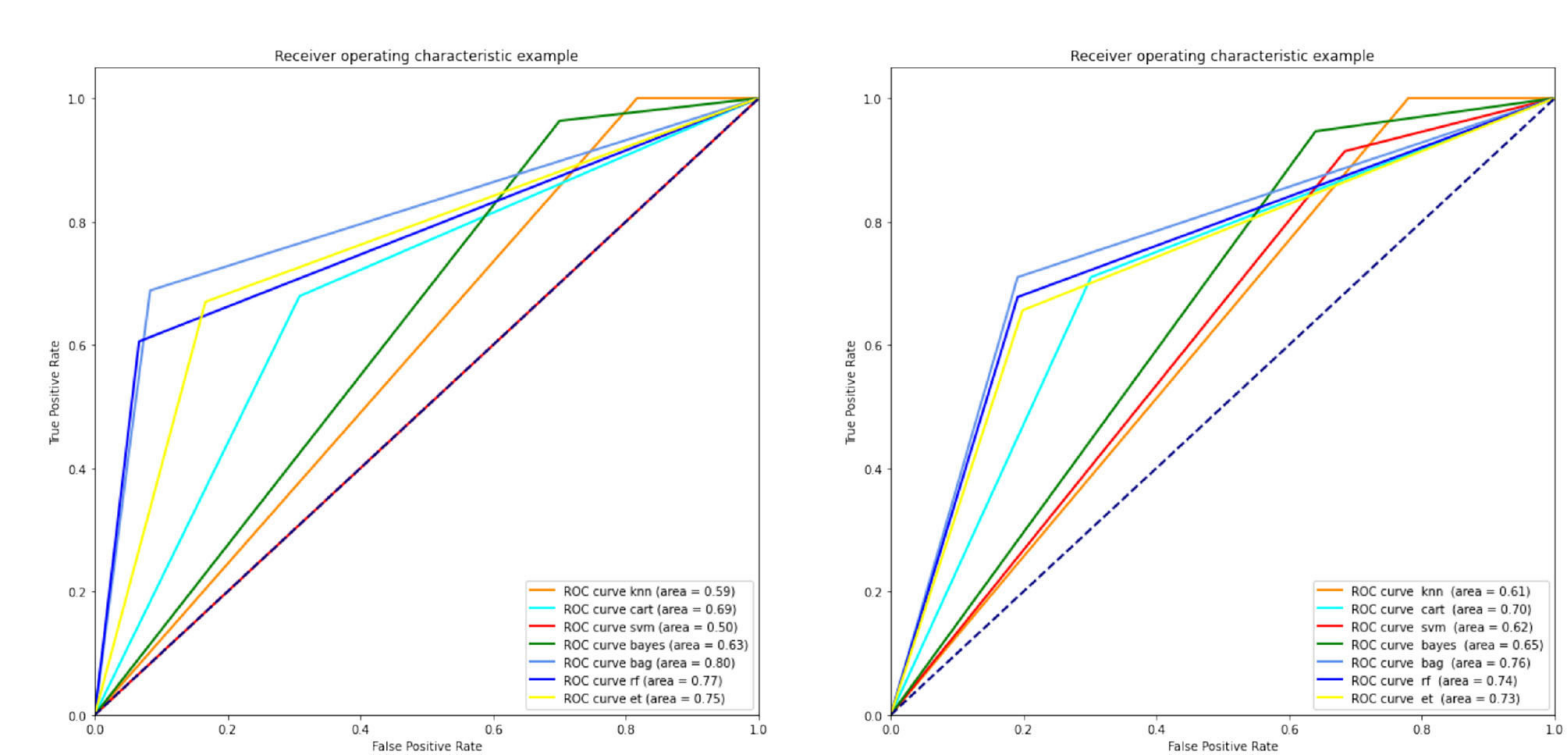
ROC curves of conventional ML classifiers. ML, machine language; ROC, receiver operating characteristic.

According to Table 8, the bagging conventional classification algorithms for EEG signals normalized using *z*-score normalization resulted in the maximum accuracy. Figure 5 shows the ROC curves for ML classification algorithms with different normalizations of EEG signals. The figure on the left shows the results of ML classification methods with z-score normalization; additionally, the ROC curves for ML classification algorithms with *z*-score + L2 normalization is presented in the figure on the right.

We also employed several DL architectures based on CNNs and LSTMs (Goodfellow et al., 2016), and the combination of both convolutions and LSTM layers. Namely, three CNNs, two LSTMs, and two CNN-LSTM networks (see Tables 1–7 for the concrete architecture of these networks) were studied. We also analyzed the relevance of using three different activation functions (ReLU, Leaky ReLU, and seLU), and the impact of z-score normalization. To avoid overfitting, we applied two regularization techniques that are Dropout (Goodfellow et al., 2016) and weight regularization (Goodfellow et al., 2016). In particular, dropout was applied after each convolutional and LSTM layer using a dropout value of 0.5, and after dense layers using a dropout value of 0.25. Weight regularization was employed in all the convolutional, LSTM, and dense layers of our architectures using L2 regularization with a value 0.01. The final selected values for batch size and hyperparameters of our networks are all available in Table 9. All the experiments were conducted using the Keras library (Gulli and Pal, 2017) and using a GPU NVidia RTX2080 Ti.

**Table 9:** The final selected values for batch size and hyperparameters of the proposed DL networks.

**Networks**	**Epochs**	**Batch size**	**Learning rate**
CNN-1	32	10	0.01
CNN-2	32	10	0.01
CNN-3	32	10	0.01
LSTM-1	30	16	0.01
LSTM-2	30	16	0.01
CNN-LSTM 1	50	128	0.01
CNN-LSTM 2	50	128	0.01

*CNN, convolutional network; DL, deep learning; LSTM, long short-term memory*.

In the following, the results obtained from the DL proposed methods for different activation functions are demonstrated in Tables 10–**12**. First, the results obtained from the proposed DL method with the Leaky ReLU activation function are demonstrated in Table 10.

**Table 10:** Performance criteria of the proposed DL methods with Leaky ReLU activation function.

**Methods**	**Leaky ReLU** **+** ***z*****-Score**				**Leaky ReLU** **+** ***z*****-Score** **+** **L2**			
	**Acc**	**Prec**	**Rec**	**AUC**	**Acc**	**Prec**	**Rec**	**AUC**
CNN-1	70.83 ± 8.76	58.12 ± 8.23	98.86 ± 1.24	80.95 ± 8.72	64.10 ± 6.68	52.17 ± 4.72	99.31 ± 0.90	86.73 ± 9.86
CNN-2	38.42 ± 0.00	38.42 ± 0.00	100.00 ± 0.00	50.00 ± 0.00	40.00 ± 1.76	39.03 ± 0.67	99.77 ± 0.45	52.21 ± 3.22
CNN-3	56.85 ± 4.17	47.24 ± 2.57	99.54 ± 0.55	67.19 ± 5.60	58.07 ± 3.77	47.93 ± 2.24	100.00 ± 0.00	82.73 ± 9.98
LSTM-1	83.32 ± 2.55	73.64 ± 3.41	88.63 ± 6.66	91.03 ± 2.02	72.31 ± 8.37	56.03 ± 29.3	51.59 ± 29.76	74.52 ± 12.28
LSTM-2	79.91 ± 9.00	72.12 ± 11.82	85.68 ± 5.90	86.90 ± 8.10	76.68 ± 6.51	70.79 ± 9.95	76.82 ± 23.80	80.30 ± 9.38
CNN-LSTM 1	74.06 ± 19.9	65.83 ± 27.45	58.40 ± 32.91	78.32 ± 20.99	94.76 ± 5.94	90.95 ± 10.6	98.86 ± 1.24	99.73 ± 0.21
CNN-LSTM 2	79.04 ± 12.2	71.51 ± 25.93	58.40 ± 36.37	85.79 ± 16.62	**97.73** **±** **1.39**	96.35 ± 3.55	97.95 ± 1.32	99.71 ± 0.15

*AUC, area under the curve; CNN, convolutional network; DL, deep learning; LSTM, long short-term memory*.

*The bold values provide the highest accuracy the method compared other methods*.

As indicated in Table 10, the second proposed CNN-LSTM model with the Leaky ReLU activation function and combined normalization of *z*-score with L2 could obtain the maximum accuracy. Table 11 presents the results obtained from the proposed DL method with the seLU activation function.

**Table 11:** Performance criteria of the proposed DL methods with seLU activation function.

**Methods**	**seLU** **+** **z-Score**				**seLU** **+** ***z*****-Score** **+** **L2**			
	**Acc**	**Prec**	**Rec**	**AUC**	**Acc**	**Prec**	**Rec**	**AUC**
CNN-1	61.65 ± 4.89	50.49 ± 3.98	95.90 ± 4.22	69.50 ± 4.06	65.67 ± 5.95	53.71 ± 5.05	94.31 ± 6.14	75.12 ± 5.90
CNN-2	57.90 ± 2.48	32.43 ± 19.4	59.77 ± 46.99	56.51 ± 11.79	58.42 ± 5.43	38.38 ± 19.4	51.36 ± 44.52	58.17 ± 8.82
CNN-3	62.09 ± 4.43	50.71 ± 3.11	93.18 ± 11.94	69.17 ± 3.67	66.46 ± 4.20	54.76 ± 4.37	88.18 ± 12.48	76.09 ± 4.23
LSTM-1	74.84 ± 5.05	64.48 ± 5.57	77.50 ± 9.15	82.90 ± 5.55	70.13 ± 8.80	57.07 ± 16.6	58.86 ± 29.15	72.11 ± 13.72
LSTM-2	**83.58** **±** **0.81**	74.99 ± 1.81	86.13 ± 3.16	91.06 ± 0.52	79.65 ± 6.27	72.75 ± 10.1	79.31 ± 8.36	86.43 ± 5.56
CNN-LSTM 1	59.73 ± 1.47	41.14 ± 6.92	8.40 ± 3.18	50.95 ± 2.38	58.42 ± 3.39	48.12 ± 2.10	99.73 ± 0.45	89.44 ± 1.57
CNN-LSTM 2	59.65 ± 3.02	43.78 ± 8.60	10.90 ± 3.26	61.16 ± 5.17	57.64 ± 1.68	47.59 ± 1.00	100.00 ± 0.00	87.08 ± 3.84

*AUC, area under the curve; CNN, convolutional network; DL, deep learning; LSTM, long short-term memory*.

Table 11 indicated that the second proposed LSTM method could result in maximum accuracy. The results of all proposed DL models with the ReLU activation function and *z*-score and L2 normalizations are presented in Table 12.

**Table 12:** Performance criteria of the proposed DL methods with ReLU activation function.

**Methods**	**ReLU** **+** **z-Score**				**ReLU** **+** **z-Score** **+** **L2**			
	**Acc**	**Prec**	**Rec**	**AUC**	**Acc**	**Prec**	**Rec**	**AUC**
CNN-1	93.27 ± 1.31	90.15 ± 4.60	93.18 ± 5.18	97.80 ± 0.35	92.66 ± 1.39	92.01 ± 2.57	88.86 ± 6.15	97.40 ± 0.60
CNN-2	84.80 ± 11.7	65.18 ± 32.79	78.63 ± 39.34	88.80 ± 19.40	84.80 ± 11.7	89.25 ± 2.55	85.84 ± 9.18	88.63 ± 8.71
CNN-3	93.97 ± 2.33	89.16 ± 5.34	96.59 ± 2.87	97.74 ± 0.85	93.18 ± 1.25	89.33 ± 5.17	94.09 ± 4.21	98.04 ± 0.23
LSTM-1	79.03 ± 3.92	69.71 ± 6.01	82.95 ± 4.711	87.76 ± 3.26	71.79 ± 7.83	67.12 ± 10.3	57.72 ± 28.8	73.71 ± 11.48
LSTM-2	71.79 ± 8.72	50.58 ± 26.85	70.45 ± 35.26	77.31 ± 14.52	71.0 ± 12.16	69.48 ± 14.5	68.18 ± 31.3	76.37 ± 12.46
CNN-LSTM 1	93.71 ± 0.71	89.09 ± 2.505	95.45 ± 1.901	96.37 ± 0.62	98.07 ± 1.47	96.01 ± 3.91	99.31 ± 0.55	99.88 ± 0.11
CNN-LSTM 2	94.76 ± 1.23	90.79 ± 1.914	96.14 ± 1.541	97.29 ± 0.50	**99.25** **±** **0.25**	98.33 ± 3.33	98.86 ± 1.24	99.73 ± 0.35

*AUC, area under the curve; CNN, convolutional network; DL, deep learning; LSTM, long short-term memory*.

*The bold values provide the highest accuracy the method compared other methods*.

**Figure 6:**
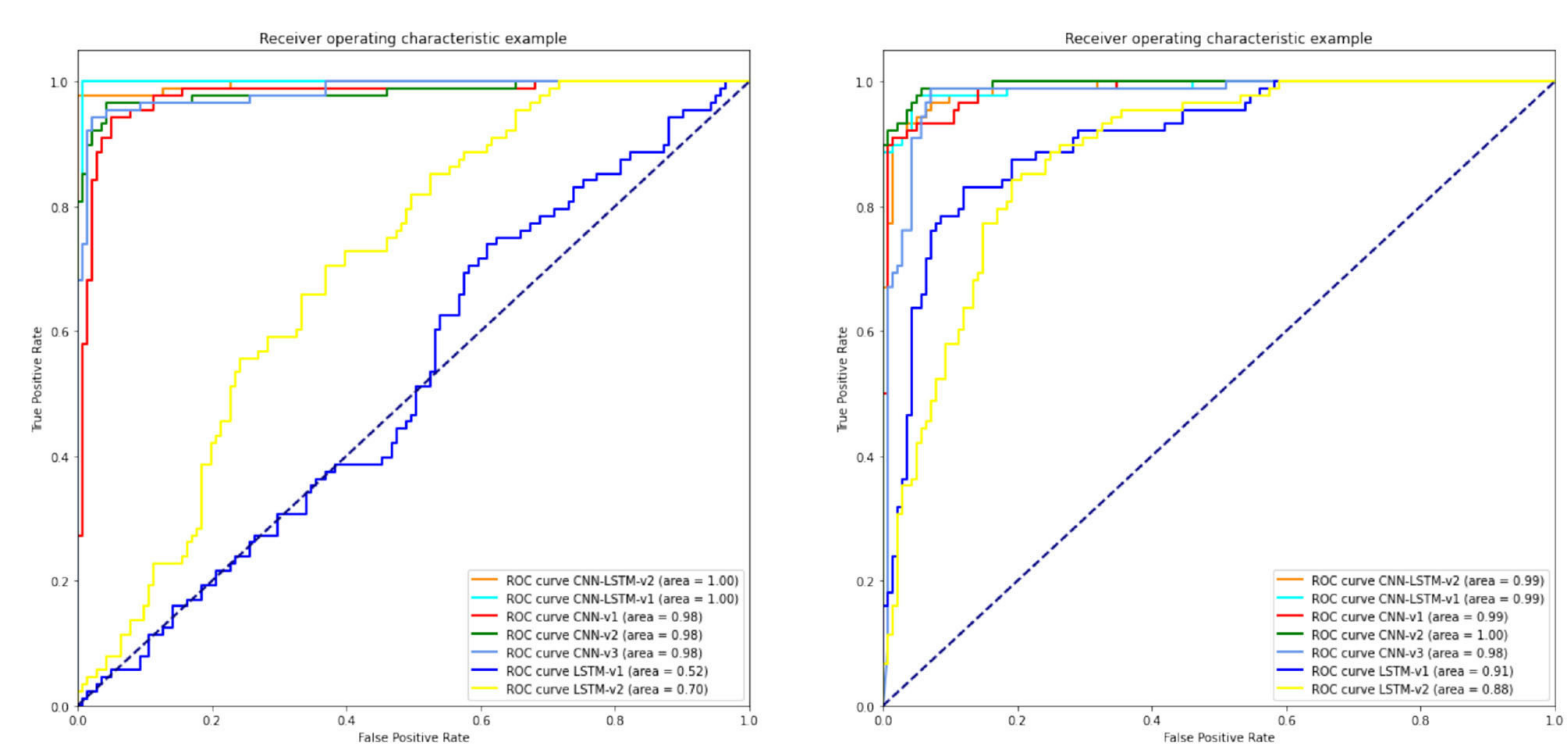
ROC curves of DL methods with ReLU activation function and *z*-score + L2 normalization. DL, deep learning; ROC, receiver operating characteristic.

**Figure 7:**
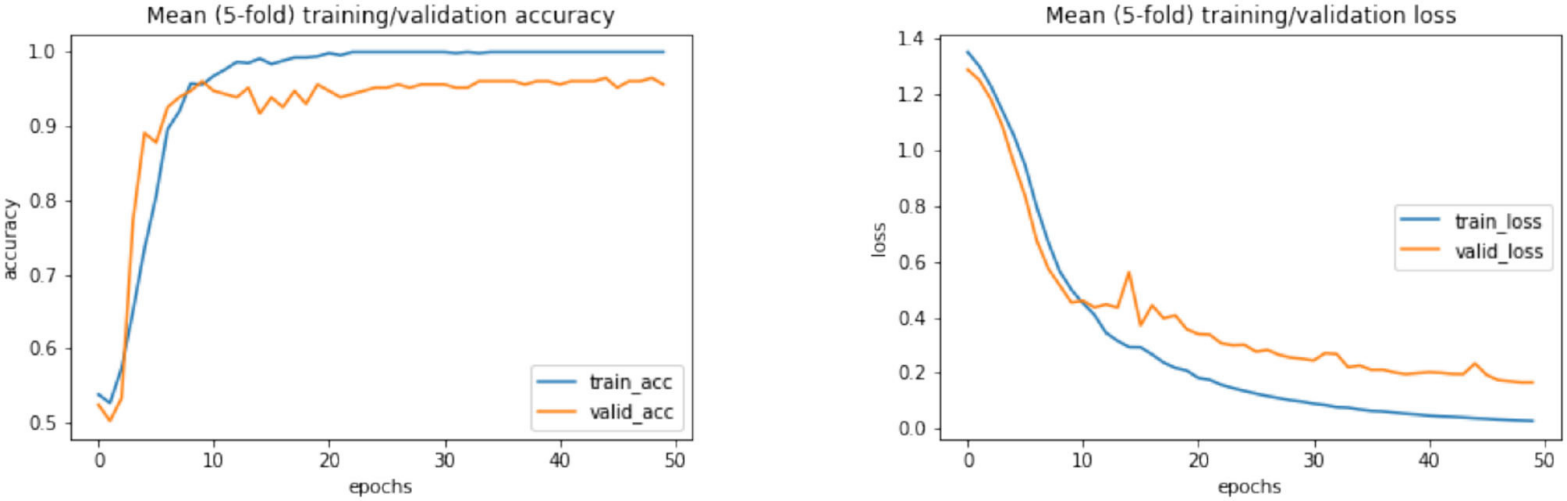
Learning curves of CNN-LSTM method with ReLU activation function and *z*-score normalization. CNN, convolutional network; LSTM, long short-term memory.

**Figure 8:**
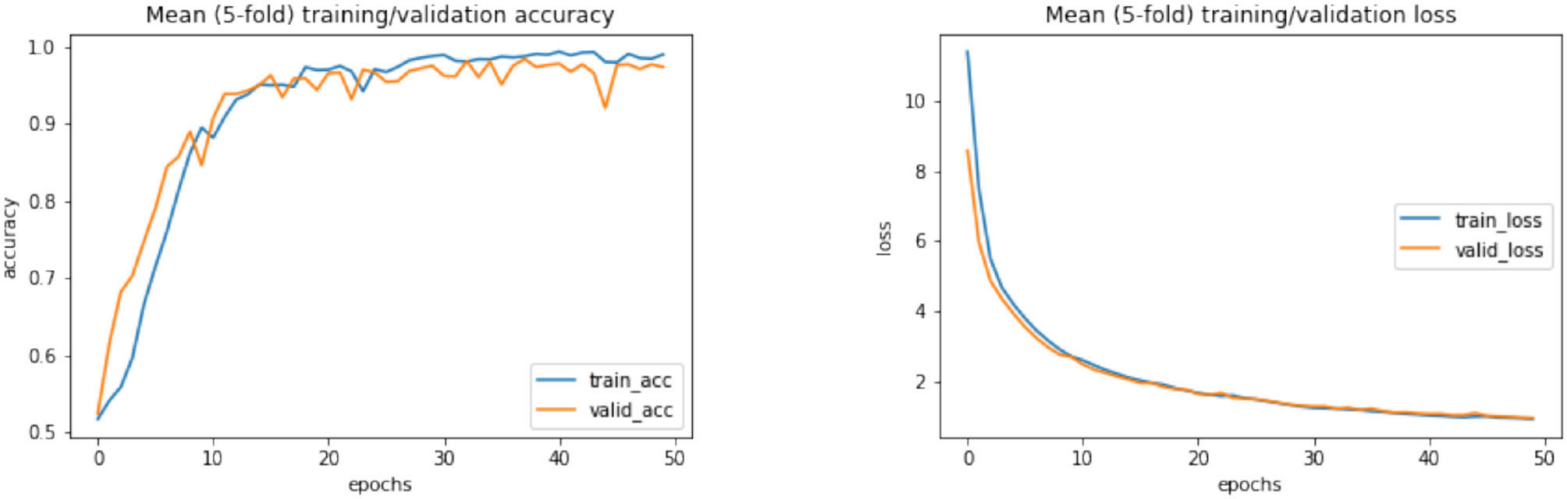
Learning curves of CNN-LSTM method with ReLU activation function and *z*-score + L2 normalization. CNN, convolutional network; LSTM, long short-term memory.

According to Table 12, it can be seen that compared to all classification methods with different activation functions, the second proposed CNN-LSTM model with ReLU activation function and combined normalization technique of *z*-score and L2 could lead to the maximum accuracy. In the following, the ROC diagrams for the DL models with ReLU activation functions and *z*-score and *z*-score + L2 normalization methods are drawn in Figure 6. Firstly, on the left of Figure 6, the results of the DL algorithms with *z*-score + L2 normalization are presented. Also, the ROC curves for DL algorithms with the *z*-score normalization for EEG signals is shown in the right of Figure 6. Furthermore, learning curves of the CNN-LSTM method with ReLU activation and *z*-score normalization and also with *z*-score + L2 normalization are shown in Figures 7, 8, respectively.

**Figure 9:**
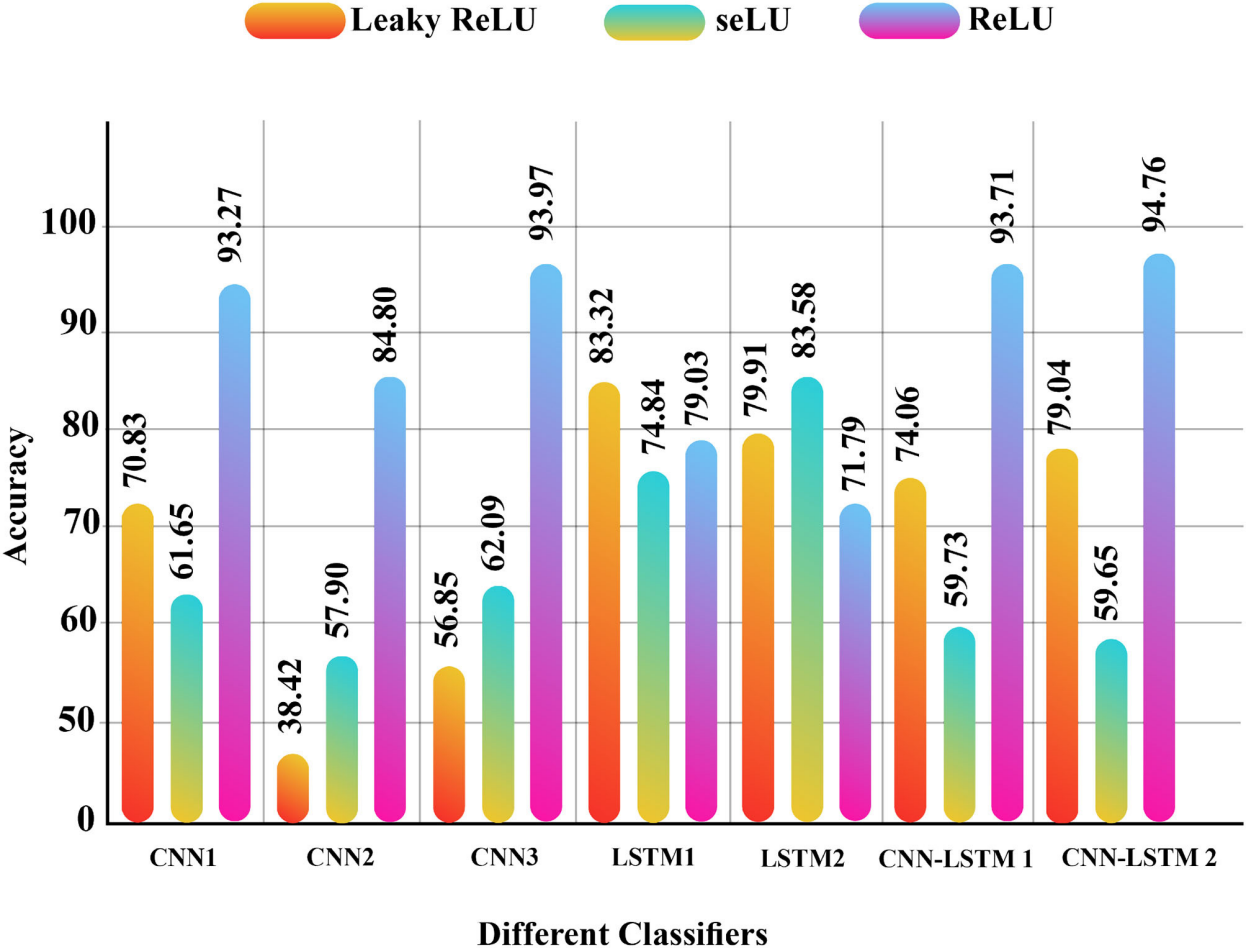
Results for different proposed DL methods with different activation functions and *z*-score normalization. DL, deep learning.

**Figure 10:**
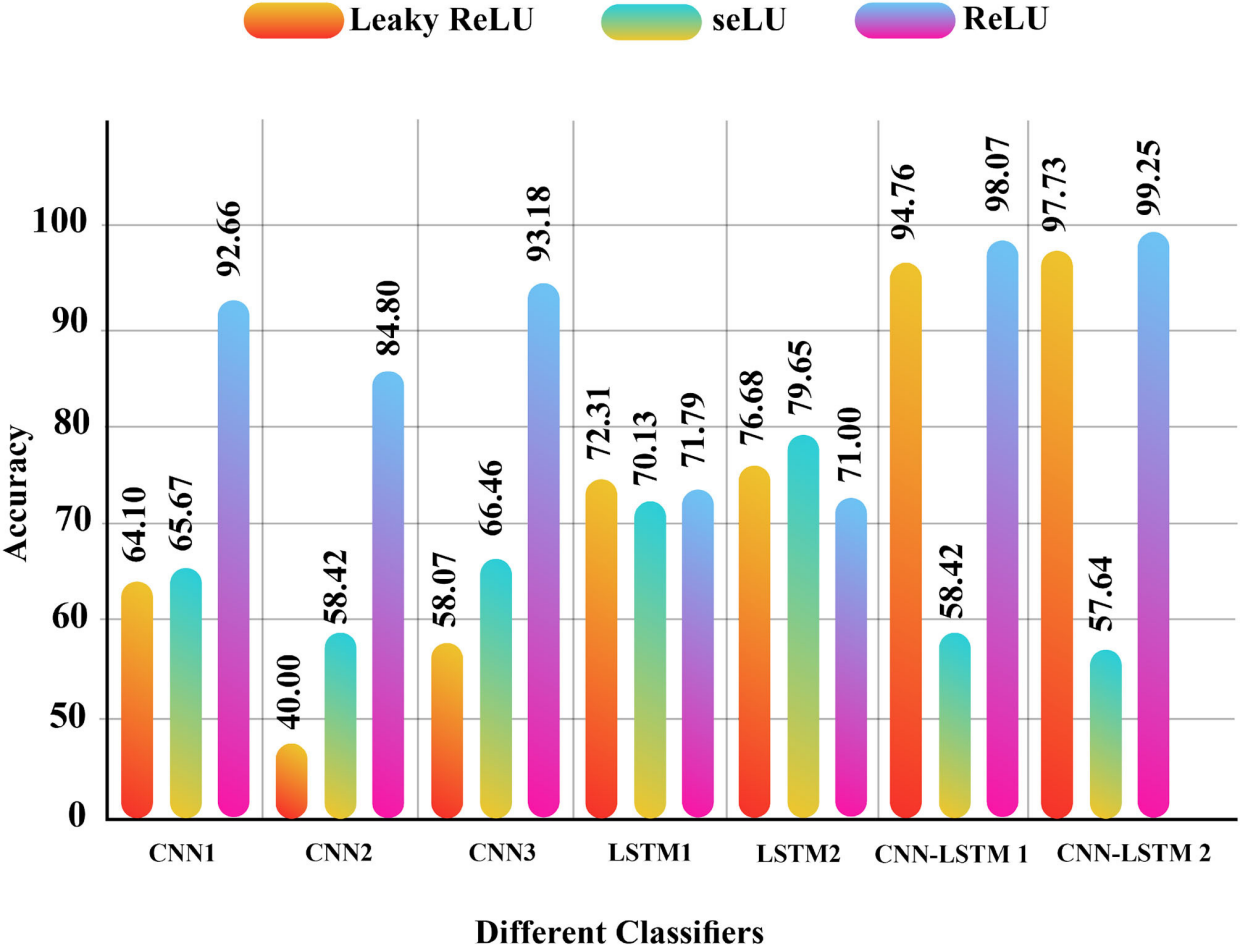
Results for different proposed DL methods with different activation functions and *z*-score with L2 normalization. DL, deep learning.

The simulation results of the proposed models for SZ diagnosis *via* EEG signals were investigated in this section. Compared to all DL and conventional ML methods, the CNN-LSTM models with 13 layers have higher accuracy and efficiency among the proposed methods. Selecting the number of layers in this model and the type of the activation functions are presented in this research for the first time, which is the novelty of the article. Besides, simultaneously using *z*-score and L2 normalizations along with the proposed CNN-LSTM model is another novelty of this article. Figure 9 shows the DL models with different activation functions and *z*-score normalization. Also, Figure 10 displayed the DL architectures with different activation functions and *z*-score and L2 normalization. According to Figures 9, 10, the second version of CNN-LSTM with *z*-score and L2 normalization has the best performance compared to other methods.

## Limitation of Study

The limitations of the study are investigated in this section. The available EEG datasets for SZ diagnosis consist of a limited number of cases which has made access to the tools of SZ diagnosis *via* EEG signals and DL models challenging. The dataset in this research was not used to determine the severity of the disorder but to diagnose the disorder. This dataset is unsuitable for prognosis or early diagnosis, and other appropriate datasets must be gathered for these purposes. Another limitation of this study is that the classifiers are not separately designed and compared for different age and gender groups, and other suitable datasets must be gathered for this purpose. Classifiers are of the two-class type and can become multiclass by adding the classes of brain disorders with similar symptoms to SZ.

## Conclusion, Discussion, and Future Works

SZ is a mental disorder that negatively affects brain function, causing various problems for the patient. Different screening methods have been introduced for SZ mental disorder diagnosis, among which the EEG functional imaging modality has captured the interest of neurologists and specialist physicians. SZ diagnosis *via* EEG signals has always been challenging. In recent years, various investigations into using AI techniques for SZ diagnosis and interpretation *via* EEG signals have been conducted to tackle this challenge. These methods are proposed to help physicians and neurologists with a quick and accurate diagnosis of SZ disorder *via* EEG signals.

Various AI approaches are presented for the diagnosis of SZ mental disorder *via* EEG signals. These approaches include using different conventional ML (Alizadehsani et al., 2021) techniques and also DL models (Martinez-Murcia et al., 2019; Górriz et al., 2020; Gorriz et al., 2021; Jiménez-Mesa et al., 2021). The AI models for SZ diagnosis *via* EEG signals consist of the following steps: dataset selection, preprocessing, feature extraction and selection, and classification.

In this study, the dataset consisted of EEG data of 14 normal individuals and patients with SZ (Olejarczyk and Jernajczyk, 2017). The EEG signals of this dataset are of a 10-channel type and have a sampling frequency of 250 Hz (Olejarczyk and Jernajczyk, 2017). In the preprocessing step, first, the EEG signals were divided into 25 s frames. Afterward, *z*-score and *z*-score-L2 were used for the normalization of EEG signals. In this section, each frame of EEG signals had a dimension of 19 × 6,250. It should be noted that the preprocessing of EEG signals for the DL models included two *z*-score and *z*-score-L2 normalization techniques.

Different conventional ML-based classification algorithms were used for SZ diagnosis *via* EEG signals. In this section, the normalized EEG signals were considered as features to be applied in classification algorithms. The employed classification algorithms included the following methods: SVM (Cortes and Vapnik, 1995), KNN (Cover and Hart, 1967), DT (Rokach and Maimon, 2007), naïve Bayes (Zhang, 2004), RF (Breiman, 2001), ERT (Geurts et al., 2006), and bagging (Friedman, 2001). The bagging classification *via* EEG signals normalized using *z*-score could obtain an accuracy of %81.22 ± 1.74, which is the highest accuracy compared to other classification methods.

In the following, different DL methods of SZ diagnosis *via* EEG signals were employed. The proposed DL methods in this section included three 1D-CNN architectures, two LSTM models, and ultimately two 1D-CNN-LSTM networks. Different activation functions, namely, Leaky ReLU, seLU, and ReLU were used to implement the proposed DL models. Besides, in all models, the sigmoid activation function was used for classification. The results of DL models for different normalization methods and activation functions were indicated in Tables 10–12. Among the proposed DL models, the 1D-CNN-LSTM architecture consisting of 13 layers with the ReLU activation function and *z*-score + L2 normalization could obtain an accuracy of %99.25 ± 0.25. This model is presented for the first time in this research, as this article's novelty. The comparison between the proposed 1D-CNN-LSTM model with the proposed models of the previous studies conducted on SZ diagnosis *via* EEG signals is indicated in Table 13.

**Table 13:** The proposed method compared with related works in diagnosis of schizophrenia.

**Work**	**Dataset**	**Number of cases**	**Preprocessing**	**Feature extraction and selection**	**Classifier**	**Accuracy (%)**
Siuly et al. (2020)	Kaggle	SZ:49, HC:32	EMD	Statistical Features + KW Test	EBT	89.59
Jahmunah et al. (2019)	Clinical	SZ:14, HC:14	Filtering	Non-linear Features + *t*-Test	SVM-RBF	92.90
Devia et al. (2019)	Clinical	SZ:11, HC:9	Filtering	ERP Features	LDA	71.00
Prabhakar et al. (2020)	Clinical	SZ:14, HC:14	ICA	Isomap + Optimization Methods	Adaboost Methods	98.77
Alimardani et al. (2018)	Clinical	SZ:23, HC:23	NA	Statistical features of SSVEPs + Fisher's Score	KNN	91.30
Li et al. (2019)	Clinical	SZ:19, HC:23	Filtering	SPN features	SVM	90.48
Prasad et al. (2013)	Clinical	SZ:5, HC:5	NA	Different Methods	Logistic Regression	
Luo et al. (2020)	Clinical	Different Cases	Interpolation algorithms	Microstate Features	RF	NA
Shim et al. (2016)	Clinical	SZ:34, HC:34	Filtering	Sensor-level and source-level features + Fisher's Score	SVM	88.24
Shalbaf et al. (2020)	Public Dataset	SZ:14, HC:14	Filtering	ResNet-18	SVM	98.60
Aristizabal et al. (2020)	Clinical	Sz:65/HC;40SZ:65/HC:45SZ:65/ HC:57	NA	CNN+LSTM	Sigmoid	72.54
Sun et al. (2021)	Clinical	SZ:54, HC:55	Filtering	CNN-LSTM	Softmax	99.22
Phang et al. (2019a)	Public Data	SZ:45, HC:39	NA	MDC-CNN	Softmax	93.06
Chu et al. (2017)	Clinical	SZ:40, HC:40	Ocular correction algorithm-filtering	CNN	RF	99.20
Oh et al. (2019)	Clinical	SZ:14, HC:14	*z*-score Normalization	CNN	Softmax	89.59
Naira and Alamo (2019)	NNCI	SZ:45, HC:39	Pearson Correlation Coefficient (PCC)	CNN	Softmax	90.00
Sharma et al. (2021)	Clinical	SZ:21, HC:24	Filtering	CNN-LSTM	Sigmoid	99.10
Singh et al. (2021)	NNCI	SZ:45, HC:39	Filtering	CNN-LSTM	Sigmoid	98.56
Phang et al. (2019b)	NNCI	SZ:45, HC:39	NA	DBN	Softmax	95.00
Proposed Method	Public Dataset	SZ:14, HC:14	Filtering, Normalization	1D CNN-LSTM	Sigmoid	**99.25**

*CNN, convolutional network; EBT, ensemble bagged tree; EMD, empirical mode decomposition; KNN, k-nearest neighbor; LDA, linear discriminant analysis; LSTM, long short-term memory; RBF, radial basis function; SPN, solitary pulmonary nodules; SSVEPs, steady-state visual evoked potentials; SVM, support vector machine; ERP, Event-related potentials; ICA, Independent component analysis; MDC-CNN, Multi-domain connectome CNN; DBN, Deep belief network. The bold values provide the highest accuracy the method compared other methods*.

As shown in Table 13, the model proposed in this research could obtain higher accuracy compared to a vast majority of conducted studies. The proposed model can be implemented on special software and hardware platforms for quick SZ diagnosis *via* EEG signals and may be employed as an assistant diagnosis method in hospitals.

In the following, some future investigations into SZ diagnosis *via* EEG signals are presented. The CNN-AE models can be employed for SZ diagnosis *via* EEG signals as the first future work. Several researchers indicate that CNN-AE models are highly efficient in neural disorders *via* EEG signals (Shoeibi et al., 2021a). As mentioned in the section of limitation of the study, the dataset used in this study is for SZ disorder diagnosis. However, providing EEG datasets for SZ disorder diagnosis can be of paramount importance for future investigations. One of the future works is to provide classification models based on DL for different age and gender groups, which requires researchers to have access to relevant data.

Another future work is using a combination of conventional ML and DL models for SZ diagnosis such that different non-linear features are extracted from EEG signals first. Afterward, the features are extracted from raw EEG signals by DL models. Ultimately, manual and DL features are combined, and the classification is carried out. Graph models based on DL are one of the new fields in diagnosing brain disorders. Accordingly, in future works, using graph models based on DL can be suitable for SZ diagnosis *via* EEG signals (Cao et al., 2016).

## Data Availability Statement

The original contributions presented in the study are included in the article/supplementary material, further inquiries can be directed to the corresponding author/s.

## Author Contributions

AS, NG, and JH: methodology. AS, YK, and JH: software. AS and JH: validation. AS, RA, and JG: formal analysis. AS, DS, and PM: resources. AS and NG: writing—original draft preparation. AS, NG, and RA: writing—review and editing. AS, Y-DZ, SN, and YK: visualization. All authors contributed to the article and approved the submitted version.

## Conflict of Interest

The authors declare that the research was conducted in the absence of any commercial or financial relationships that could be construed as a potential conflict of interest.

## Publisher's Note

All claims expressed in this article are solely those of the authors and do not necessarily represent those of their affiliated organizations, or those of the publisher, the editors and the reviewers. Any product that may be evaluated in this article, or claim that may be made by its manufacturer, is not guaranteed or endorsed by the publisher.
